# Synthetic tactile perception induced by transcranial alternating-current stimulation can substitute for natural sensory stimulus in behaving rabbits

**DOI:** 10.1038/srep19753

**Published:** 2016-01-21

**Authors:** Javier Márquez-Ruiz, Claudia Ammann, Rocío Leal-Campanario, Giulio Ruffini, Agnès Gruart, José M. Delgado-García

**Affiliations:** 1 Division of Neurosciences, Universidad Pablo de Olavide, 41013-Seville, Spain; 2 Starlab Barcelona SL, Tibidabo 47, 08035-Barcelona, Spain

## Abstract

The use of brain-derived signals for controlling external devices has long attracted the attention from neuroscientists and engineers during last decades. Although much effort has been dedicated to establishing effective brain-to-computer communication, computer-to-brain communication feedback for “closing the loop” is now becoming a major research theme. While intracortical microstimulation of the sensory cortex has already been successfully used for this purpose, its future application in humans partly relies on the use of non-invasive brain stimulation technologies. In the present study, we explore the potential use of transcranial alternating-current stimulation (tACS) for synthetic tactile perception in alert behaving animals. More specifically, we determined the effects of tACS on sensory local field potentials (LFPs) and motor output and tested its capability for inducing tactile perception using classical eyeblink conditioning in the behaving animal. We demonstrated that tACS of the primary somatosensory cortex vibrissa area could indeed substitute natural stimuli during training in the associative learning paradigm.

The study and development of brain-computer interfaces (BCI) constitutes an exciting field in neuroscience^1^,^2^,^3^,^4^. The use of brain-derived signals for controlling external devices and the possibility of doing it using non-invasive tools has promoted the BCI application to neurological rehabilitation^5^,^6^, communication, and motor control^1^,^2^,^3^,^7^. Intracortical microstimulation of the sensory cortex has been used for closing the loop allowing for computer-brain interfaces (CBI)^8^,^9^,^10^,^11^,^12^. Although these studies call for a major role of sensory cortical prostheses in restoring neurological functions and establishing new communication paradigms, its invasive nature seriously limits its use in human subjects. In contrast, two non-invasive methods for brain stimulation, transcranial magnetic stimulation (TMS)^13^ and transcranial current stimulation (tCS)^14^, have recently revolutionized the functional study of normal and pathological human brains. The successful application of these two non-invasive techniques for CBI could exponentially increase the number of potential applications where computer feedback is needed. For example, conscious transmission of information between human brains through neuronavigated robotized TMS has been recently demonstrated^15^. In addition, seizure-triggered feedback transcranial electrical stimulation has been successfully used in a rodent model of generalized epilepsy for reducing spike-and-wave episodes^16^. The particular advantages of tCS, a low-cost, painless and well-tolerated^17^,^18^ technique capable of being administered by portable devices not requiring complex instrumental manipulation, make it particularly interesting for CBI purposes.

It is known that direct-current (DC) stimulation of the cerebral cortex has noticeable effects on behavioral and cognitive processes in humans^14^,^17^,^19^ and animals^20^. Although the neural basis mediating tCS effects is partly unknown, it is assumed that the externally applied electric field forces the displacement of intracellular ions (which mobilize to cancel the intracellular field), altering the neuron’s internal charge distribution and modifying the transmembrane potential difference^21^. Interestingly, these changes persist for several minutes after the DC stimulus offset^22^ sharing some molecular mechanisms with long-term plasticity^23^,^24^,^25^.

Although the number of basic and clinical studies using tDCS has grown exponentially in the last decade^17^,^18^ the effects of tACS on the cerebral cortex are now starting to be explored^26^. tACS seems to interact with ongoing cortical oscillations enhancing, or reducing, the activity at specific electrocortical frequencies and their potentially related functions^27^,^28^,^29^,^30^,^31^,^32^,^33^. Interestingly, tACS also constitutes a useful approach for the study of perception processes and sensorimotor integration at the cerebral cortical level^31^. Feurra *et al*. (2011) reported that tACS applied over the human somatosensory cortex can evoke tactile sensations when alpha and high-gamma stimulation frequencies are used^31^. In support of human studies, extracellular recordings in behaving rats have shown that tACS reliably entrained neurons in widespread neocortical and hippocampal areas^34^.

In the present study, we explore the use of tACS in behaving rabbits as a non-invasive brain stimulation technique for synthetic tactile perception. Specifically, we determined the effects of tACS on sensory LFPs and motor output and tested tACS’ capability for inducing tactile perception with the help of classical eyeblink conditioning tasks. We demonstrated that tACS of the primary somatosensory cortex (SI) vibrissa area can be used for the substitution of natural sensory stimulus during associative learning.

## Results

### Low-frequency tACS induces immediate effects on LFP evoked in the SI by natural whisker stimulation

**Figure 1:**
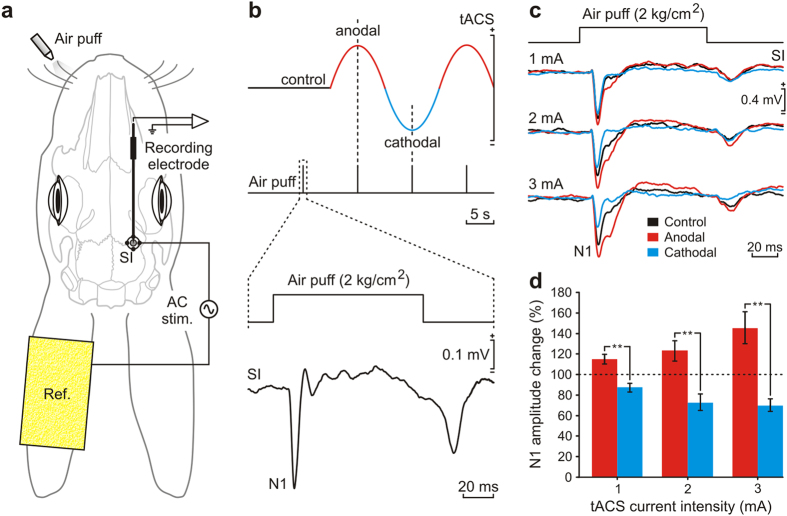
Modulation of sensory LFPs by low-frequency tACS application. (**a**) Experimental design with indication of electrode location over the SI cortex for tACS (AC stim.) and of the chronically implanted tungsten recording electrode. tACS was applied between silver electrodes implanted over the SI cortex and a large (35 cm^2^) sponge electrode (Ref.) placed on the contralateral ear. (**b**) From top to bottom are illustrated sinusoidal signal corresponding to tACS (0.05 Hz) applied during SI recording together with simultaneous air puff pulses (100 ms in duration, 2 kg/cm^2^ in pressure) presented before tACS (control) and in coincidence with the peak (anodal) or the trough (cathodal) of the sinusoidal signal. A representative average (n = 21) of LFPs evoked in the vibrissa SI cortex by air-puff stimulation of the contralateral whisker pad in control conditions is illustrated at the bottom. (**c**) Representative mean average of LFPs (n = 30) evoked in the vibrissa SI cortex by air-puff stimulation of the contralateral whisker pad in controls (black recordings) and during the application of the air puff in coincidence with anodal peaks (red recordings) or cathodal troughs (blue recordings) at increasing intensities (1, 2, and 3 mA). (**d**) Changes in amplitude of the N1 component of air-puff-evoked LFPs in the presence of anodal peaks (red histograms) or cathodal troughs (blue histograms) at increasing intensities. **P < 0.01, n = 5, one-way ANOVA. Horizontal dotted lines indicate control values. Error bars represent SEM. Calibration as indicated.

In a first series of experiments, we determined whether slow tACS applied to the somatosensory cortex could modify the characteristics of LFPs simultaneously evoked in the SI vibrissa area of behaving rabbits. Animals (n = 5) were prepared for the chronic recording of LFPs evoked in the SI by air-puff stimulation of the contralateral whisker pad and for the simultaneous application of low-frequency tACS (Fig. 1a). Whisker stimulation evoked a short-latency (14.7 ± 1.3 ms; mean ± SEM; n = 5) negative LFP (N1, Fig. 1b), followed by late positive and negative components and by a smaller LFP evoked at the end of the air puff. The amplitude of air-puff-evoked LFPs was dependent on the sensorial stimulus intensity and on the recording site across somatosensory cortical layers^25^. To determine changes induced by tACS application on LFPs, whisker stimulation was presented every 10 s during control conditions (when no current was injected) and triggered by peaks (anodal condition, with the current flowing into the cortical surface) and troughs (cathodal condition) of the sinusoidal signal corresponding to tACS (0.05 Hz). The mean average of LFP evoked in each one of these conditions was calculated for each animal at different (1 mA, 2 mA, and 3 mA, peak-to-peak amplitudes) tACS intensities. As shown in Fig. 1c, the amplitude of the N1 component of the LFP was augmented by the simultaneous presence of anodal tACS peaks (red trace in Fig. 1c) and reduced by cathodal tACS troughs (blue trace in Fig. 1c). Indeed, the N1 component of the evoked LFP was significantly increased in amplitude up to a maximum of 145.3 ± 15.6% (red bars in Fig. 1d) in response to anodal tACS peaks (n = 5, *P* ≤ 0.01; one-way ANOVA). In contrast, during cathodal tACS troughs the amplitude of the N1 component decreased to a minimum value of 70.1 ± 6.0% (blue bars in Fig. 1d) as compared with N1 values collected from controls and during the presentation of anodal tACS peaks (n = 5, *P* ≤ 0.01; one-way ANOVA). Thus, low-frequency tACS applied to the SI was capable of increasing or decreasing the amplitude of LFPs depending on the direction of the concurrently administered transcranial current.

### tACS frequency determines the appearance of motor evoked responses when applied over the motor cortex

**Figure 2:**
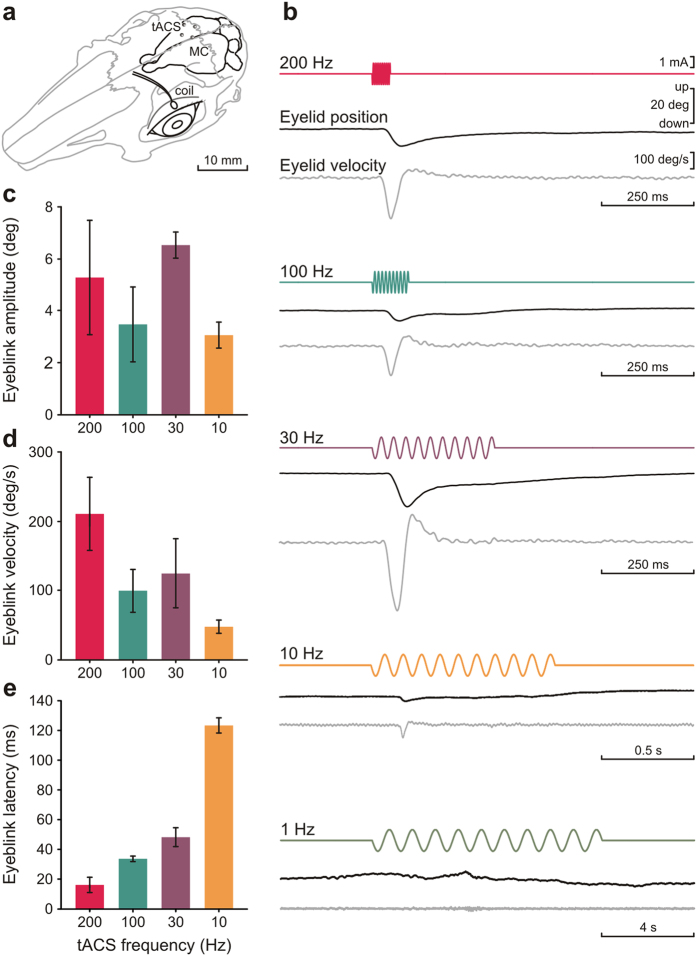
Supra-threshold effects of tACS when applied over the motor cortex. (**a**) Experimental design showing the 4 silver ball electrodes placed over the motor cortex (MC) for transcranial current stimulation. tACS was applied at different frequencies (0.01–200 Hz) maintaining the same current intensity and number of cycles (10 cycles). A five-turn coil was placed in the upper eyelid for eyelid position and velocity recording by means of the magnetic search coil technique. (**b**) Horizontal traces represent from top to bottom the transcranially applied current intensity and eyelid position and velocity recording for each one of the used frequencies (0.1 and 0.05 Hz data not shown) from a representative animal. Calibration as indicated. (**c–e**) Histograms showing amplitude (**c**), peak velocity (**d**) and latency (**e**) for eyeblinks evoked by tACS (200 Hz, 100 Hz, 30 Hz, and 10 Hz; n = 3 animals). Error bars represent SEM.

In a second step, we tested the use of tACS at slow and fast frequencies for inducing tactile perception by transcranial SI stimulation. Unlike tDCS, where sub-threshold modulation of spontaneous cortical activity has been proposed, tACS capability for inducing tactile perception will depend on its effectiveness to induce action potentials in the SI neurons (either by direct trans-membrane potential disturbance or network resonance modulation). With the aim of demonstrating supra-threshold effects we recorded eyelid position in the rabbits (n = 3) during tACS (2–3 mA peak-to-peak amplitude) of the contralateral motor cortex corresponding to the eyelid at different frequencies (0.05 Hz, 0.1 Hz, 1 Hz, 10 Hz, 30 Hz, 100 Hz and 200 Hz; 10 cycles). Figure 2 shows the experimental design (Fig. 2a) and eyelid position and velocity recorded during tACS for a representative animal (Fig. 2b). tACS of the eyelid-corresponding motor cortex induced a noticeable eyeblink in the rabbits for 30, 100 and 200 Hz stimulation frequencies being less evident when 10 Hz stimulation was used. Although no other motor effects were controlled for, tACS-induced eyeblinks were accompanied in some cases by weak movement of other face muscles and of the whiskers. No eyeblink was induced at 1, 0.1 or 0.05 Hz stimulation frequencies. As shown in Fig. 2c,d, the maximum eyeblink amplitude was obtained for 30 Hz tACS stimulation, this being 6.5 ± 0.5 deg (n = 3), whilst the maximum velocity was acquired at 200 Hz reaching 210.1 ± 54.1 deg/s (n = 3). Eyeblink latency values (Fig. 2e) showed a continuous increase from higher to lower frequencies, the minimum value for 200 Hz was 15.6 ± 5.8 ms (n = 3), whilst for 10 Hz latency value was delayed to 123.1 ± 6.2 ms (n = 3). Importantly, these results suggest that the sub- or supra-threshold nature of tACS-associated effects depends on the frequency of the applied current. The modulatory effects observed in the SI during 0.05 Hz tACS support this conclusion.

### tACS of the SI vibrissa area can substitute for whisker stimulation during classical eyelid conditioning

**Figure 3:**
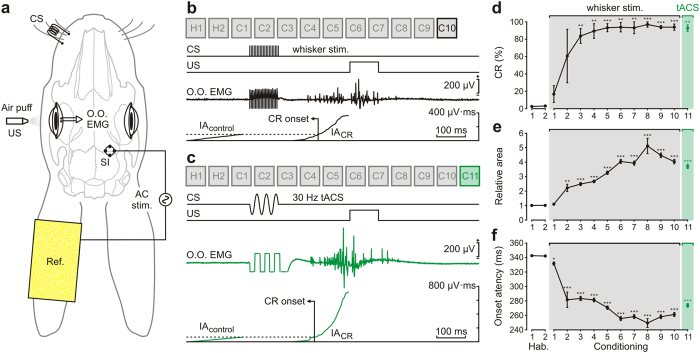
tACS of the somatosensory cortex substitute whisker stimulation during classical eyeblink conditioning. (**a**) Experimental design for tACS of primary somatosensory cortex (SI) and eyeblink conditioning. tACS was applied between 4 silver electrodes implanted over SI cortex and a large (35 cm^2^) sponge electrode (Ref.) placed on the contralateral ear. For classical conditioning animals were presented with a train (100 ms, 200 Hz) of electrical stimuli applied to the whisker pad as CS, followed 250 ms later by an air puff (100 ms, 3 kg/cm^2^) presented to the ipsilateral cornea as US. Recording electrodes were implanted in the left orbicularis oculi muscle (O.O. EMG) to evaluate the evoked CR. (**b**) Electrical whisker pad stimulation was presented as CS during conditioning sessions (C1–C10). We present from top to bottom the CS and US presentations, and a representative EMG recording collected during C10 session. For a valid CR the integrated EMG activity (IA) recorded during the CS-US interval (IA_CR_) had to overtake the integrated EMG activity recorded immediately before CS presentation (IA_control_). The CR onset was defined by the point where IA_CR_ surpassed IA_control_. (**c**) During the 11th session (C11) tACS over SI cortex corresponding to whisker pad was presented substituting the electrical whisker stimulation. From top to bottom the same than for **(b)**, illustrating a representative EMG recording from the same animal during C11 with tACS 30 Hz as CS. Calibration as indicated. (**d–f**) Evolution of CR percentage **(d)**, relative area **(e)** and the latency **(f)** of CRs during the 10 successive sessions (gray background). Relative area values were calculated as the rate between IA_CR_ and IA_control_ and latency values as the time difference between CS initiation and CR onset. Black circles represent responses to whisker stimulation, whilst green circles represent responses evoked by tACS. The number of CRs (*P* < 0.01, one-way ANOVA) and relative area (*P* < 0.01, Mann-Whitney test) increased significantly across sessions whereas the CR onset latency significantly decreased (*P* < 0.05, Mann-Whitney test) as compared with habituation sessions. Interestingly, these significant differences were maintained for the tACS-CS induced CRs (green background) (*P < 0.05; **P < 0.01; ***P < 0.001). Error bars represent SEM.

In a third series of experiments, we checked whether tACS applied over the SI was capable of inducing a tactile sensation. We tested whether a short (100 ms) sinusoidal-wave current applied to the SI could substitute for the whisker conditioned stimulus (CS) during an associative learning task. In this case, we used the EMG activity of the orbicularis oculi to compare conditioned eyeblink responses (CRs) induced by peripheral whisker-pad stimulation vs. tACS-induced CRs. The presence of CRs was determined by recording the EMG activity of the ipsilateral orbicularis oculi muscle. This experimental design allowed recording CRs and the simultaneous application of tACS to the contralateral vibrissa area of the SI (Fig. 3a). Following a previous study^8^, we used a train of pulses (100 ms, 200 Hz, <2 mA) presented to the whisker pad as CS, followed 250 ms from its end by an air puff directed at the ipsilateral cornea as US. A total of two habituation (when the CS was presented alone) and ten conditioning (including paired CS-US presentation) sessions were carried out during 12 successive days. A conditioning session lasted ~80 min and the interval between consecutive sessions was ~24 h. In order to characterize the CR we defined the CR onset as the point where the integrated activity of the CR (IA_CR_) overtook the integrated activity during the 200 ms before CS onset (control situation) (IA_control_). The CR latency was calculated as the time difference between the CS initiation and the CR onset. In order to quantify the strength of the CR, the relative area value for each CR was calculated as the ratio between IA_CR_ and IA_control_.

After ten conditioning sessions, all the animals showed a clear CR when the peripheral CS was presented (Fig. 3b). An additional conditioning session (C11) was performed in order to test whether tACS over the SI was able to mimic a sensory input similar to that evoked by whisker stimulation. Based on preliminary experiments performed in separated animals, the applied tACS consisted of a short sinusoidal wave of 100 ms in duration, 30 Hz in frequency, and intensities ranging from 3 mA to 4 mA peak-to-peak amplitudes. The EMG trace illustrated in Fig. 3c shows a representative CR after tACS stimulation at 30 Hz of the same animal illustrated in Fig. 3b. tACS-CS induced CRs similar to those observed when direct stimulation of the whisker pad was carried out.

We also analyzed the percentage, relative area, and latency of CRs obtained with the two experimental procedures. Across the training period where whisker stimulation was used as CS, animals reached asymptotic values of CRs (>80% of per session) for their learning curves by the 3rd-4th conditioning session (Fig. 3d). From the 3rd conditioning session onwards, the number of CRs increased significantly (n = 3, P < 0.01; repeated-measures ANOVA) across conditioning sessions as compared with habituation sessions. The percentage of CRs for the 10th (C10) session was 93.9 ± 3.5% (n = 3). When peripheral CS was substituted by tACS-CS in the 11th conditioning session (C11), the percentage of CRs was 92.8 ± 3.6% (n = 3), showing no statistical significant difference with respect to the previous conditioning session (n = 3, *P* = 0.826; one-way ANOVA). The analysis of the relative area of CRs and the latency to CR onset (including all eyeblink responses) across conditioning sessions also showed significant differences as compared with habituation sessions (*P* < 0.001; Friedman repeated-measures ANOVA). Thus, the relative area of CRs increased significantly from the 2nd conditioning session (*P* < 0.01; Mann-Whitney test, Fig. 3e), while CR onset latency decreased from the 1st conditioning session (*P* < 0.05; Mann-Whitney test, Fig. 3f). These significant differences were maintained for the tACS-CS-induced CRs. In order to compare the characteristics of CRs evoked by peripheral whisker stimulation and those evoked by tACS pulses, we measured the relative area and the onset latency of both types of CRs. Regarding the CR relative area, no significant (*P* = 0.082; Mann-Whitney test) difference was found between CRs evoked by peripheral whisker stimulation (4.2 ± 0.1; n = 185) and those evoked by tACS stimulation of the SI (3.9 ± 0.1; n = 181). In contrast, a small although statistically significant difference was found in the onset latency of CRs when peripheral (256.1 ± 2.5 ms; n = 185) or tACS stimulation (269.2 ± 2.3 ms; n = 181) was used (*P* ≤ 0.001; Mann-Whitney test). The values for relative area and onset latency for CRs collected from each conditioned animal are presented in Supplementary Table 1.

### Rabbits discriminate between different tACS frequencies during classical eyeblink conditioning

**Figure 4:**
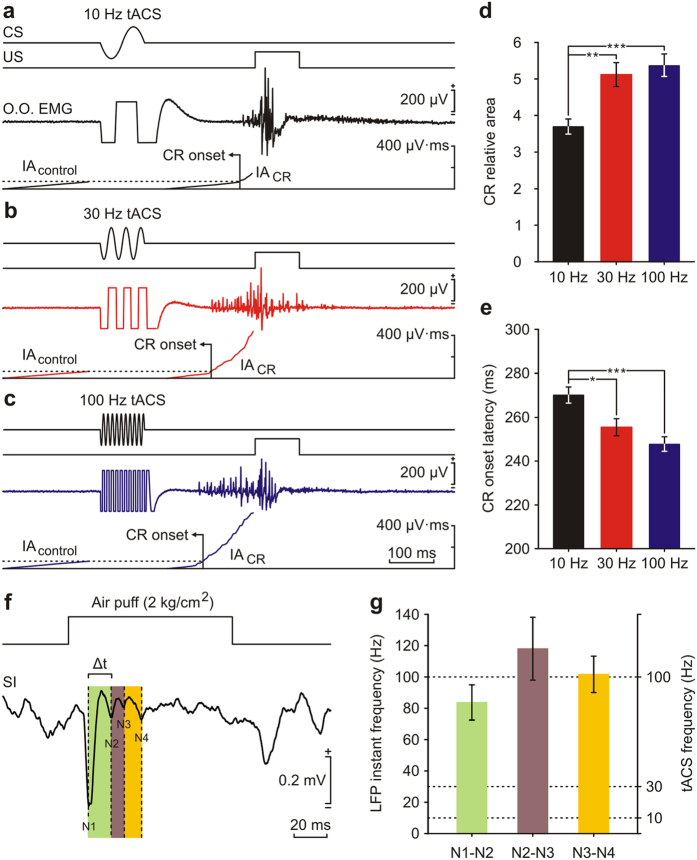
Effects of tACS frequency on the evoked CR during classical eyeblink conditioning. (**a–c**) From top to bottom CS and US presentations, a representative EMG recording from the orbicularis oculi muscle (EMG O. O.) and the corresponding integrated activity (IA) for a 10 Hz (**a**), 30 Hz (**b**), and 100 Hz (**c**) tACS pulse during the conditioning session C12 of the same animal are represented. (**d**) Histogram bars represent the relative area of CRs collected from tACS stimulation at 10 Hz (n = 60), 30 Hz (n = 64), and 100 Hz (n = 66). The relative area of evoked CRs was significantly lower for 10 Hz in comparison to 30 Hz and 100 Hz (Mann-Whitney test). (**e**) Histogram bars represent the onset latency of the CR for data collected from tACS stimulation at 10 Hz (n = 60), 30 Hz (n = 64), and 100 Hz (n = 66). Latency values were significantly higher for stimulations at 10 Hz in comparison to stimulations at 30 and 100 Hz (Mann-Whitney test). *P < 0.05; **P < 0.01; ***P < 0.001. Error bars represent SEM. Calibration as indicated. (**f**) Representative averaged LFP recording (n = 30) of the oscillatory activity evoked in the primary somatosensory cortex (SI) by air puffs presented to the contralateral whisker pad. N1 to N4 indicate the consecutive troughs (vertical dashed lines) present in the recorded LFP. (**g**) The mean averaged time between two consecutive troughs (Δt) was used to calculate the mean instant frequency corresponding to each period (n = 5). Mean instant frequency data were represented and compared with the three frequencies used for tACS (horizontal dashed lines). Error bars represent SEM.

In order to test whether animals could discriminate between different tACS frequencies, an extra conditioning session (C12) was carried out in well-trained animals. In this last session, whisker-pad stimulation was substituted by tACS stimulation at 10 Hz, 30 Hz, and 100 Hz in the three participating animals (22 stimuli for each frequency value randomly distributed). Figure 4 shows representative CRs collected during the 12th conditioning session for 10 Hz, 30 Hz, and 100 Hz (Fig. 4a–c) of tACS stimulation. As shown in Fig. 4 and Supplementary Table 2, 10 Hz tACS was able to induce CRs surpassing the established criterion, but these evoked CRs were smaller in their relative area and of larger latencies than those evoked by 30 Hz and 100 Hz tACS. Thus, the relative area of the CRs induced by 10 Hz tACS (3.7 ± 0.2; n = 60) was significantly (*P* ≤ 0.01; Mann-Whitney test) different from that of those induced by 30 Hz tACS (5.1 ± 0.3; n = 64) or 100 Hz tACS (5.4 ± 0.3; n = 66). In contrast, no statistical (*P* = 0.427) difference was observed between 30 Hz and 100 Hz tACS (Fig. 4d). Similar results were obtained for the onset latency of CRs (Fig. 4e), where values collected from 10 Hz tACS (269.9 ± 3.6 ms; n = 60) were significantly (*P* ≤ 0.05; Mann-Whitney test) higher than those collected from 30 Hz tACS (255.4 ± 3.9; n = 64) or 100 Hz tACS (247.5 ± 3.2; n = 66). No significant (*P* = 0.145) difference was observed for the onset latency of CRs between tACS of 30 Hz and that of 100 Hz (Fig. 4e).

To explore the possibility that the different results for the three frequencies used for tACS depended on physiologically evoked frequencies during natural CS presentation (i.e., peripheral whisker stimulation), we analyzed the oscillatory properties of the LFP evoked in the SI by an air puff on the contralateral whisker pad. For that, we selected those LFP recordings where oscillations after the main N1 component were clearly observed (a total of 64 LFPs in 5 animals). The latency from the air-puff onset to the different LFP components was calculated averaging the data, and resulted in 14.7 ± 1.3 ms for N1, 28.1 ± 3.9 ms for N2, 37.9 ± 5.4 ms for N3, and 48.2 ± 6.3 ms for N4 (n = 5). A representative average of LFPs (n = 10) with the associated components is presented in Fig. 4f. To compare the frequency of each one of the components with the different frequencies used for tACS stimulation, we calculated the instantaneous frequency of the components as the inverse of the duration between consecutive peaks (Δt in Fig. 4f). Instant frequencies associated to the different intervals were 83.7 ± 11.2 Hz for N1-N2, 118.0 ± 20.1 Hz for N2-N3, and 101.7 ± 11.6 Hz for N3-N4 (n = 5; Fig. 4g). Thus, the different components of LFPs evoked in the SI by whisker stimulation tended to oscillate at about 100 Hz, a frequency that proved to be suitable for tACS used as a CS.

### tACS of the SI vibrissa area can train the animals for natural sensory stimulus during classical eyelid conditioning

Considering the above results, it would be expected that: 1) animals could be entirely conditioned by using tACS-CS from the beginning of the eyeblink conditioning protocol, 2) animals conditioned by tACS-CS would respond in a conditioned manner to peripheral whisker pad stimulation (100 ms, 200 Hz), and 3) the CRs obtained from peripheral whisker pad stimulation would not differ from tACS-induced CRs.

**Figure 5:**
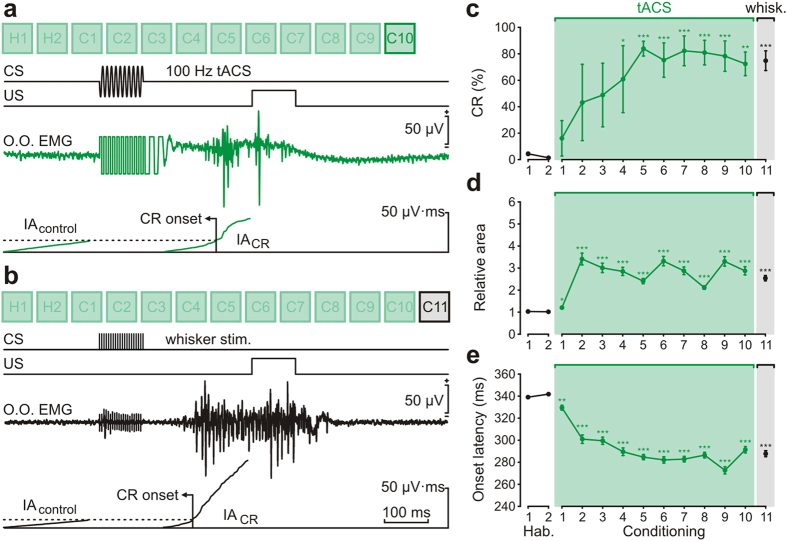
tACS of the SI vibrissa area can train the animals for natural sensory stimulus during classical eyeblink conditioning. (**a**) A total of 2 habituation and 10 conditioning sessions were carried out. tACS at 100 Hz was presented as CS during conditioning sessions C1 to C10. From top to bottom the CS and US presentations, a representative EMG recording from the orbicularis oculi muscle (O.O. EMG) and the corresponding integrated activity (IA) during C10 session are represented. (**b**) During the 11th conditioning session (C11) tACS stimulation over SI cortex corresponding to whisker pad was substituted by peripheral electrical whisker stimulation. A representative EMG recording from the same animal during C11 in response to peripheral whisker stimulation as CS is shown. Note that the evoked CR by whisker pad stimulation was similar to that evoked by tACS during the C10 session, as illustrated in (**a**). Calibration as indicated. (**c–e**) Evolution of the learning curve across conditioning. The evolution of CR percentage (**c**), relative area (**d**) and latency (**e**) of CRs during the successive conditioning sessions by using tACS as CS are presented (green background). Green circles represent responses to tACS stimulation, whilst the black circle represents responses evoked by peripheral electrical whisker stimulation (gray background). The number of CRs (*P* < 0.05; one-way ANOVA) and the relative area of CRs (*P* < 0.05; Mann-Whitney test) increased significantly across conditioning sessions whereas the latency to the CR onset significantly decreased (*P* < 0.01; Mann-Whitney test) as compared with habituation sessions. Interestingly, these significant differences were maintained for the tACS-CS induced responses (*P < 0.05; **P < 0.01; ***P < 0.001). Error bars represent SEM.

In order to test these possibilities, a fourth group of animals (n = 3) was prepared for the chronic tACS of SI as CS, whilst an air puff presented to the ipsilateral cornea was used as US. We used the same conditioning protocol and criterion as for previous eyeblink conditioning. After 10 conditioning sessions, all the animals showed a clear CR when tACS-CS was presented (Fig. 5a). As expected, the peripheral whisker pad stimulation (100 ms, 200 Hz) in an additional conditioning session (C11) induced CRs similar to those observed when tACS of SI was carried out (Fig. 5b).

As before, we also analyzed the percentage, relative area, and latency of CRs obtained with the two experimental procedures. Along the training period where tACS of SI was used as CS, animals reached asymptotic values of CR percentage (>70% of CRs per session) for their learning curves by the 5th conditioning session (Fig. 5c). From the 4th conditioning session onwards, the number of CRs increased significantly (n = 3, P < 0.05; repeated-measures ANOVA) across conditioning sessions as compared with habituation sessions. The percentage of CRs for the 10th (C10) session was 72.5 ± 9.1% (n = 3). A very similar value was obtained when peripheral CS substituted tACS-CS in the 11th conditioning session (C11): the percentage of CRs was 74.8 ± 7.6% (n = 3), showing no significant difference with respect to the previous conditioning session (n = 3, *P* = 0.852; one-way ANOVA). Regarding to the analysis of the relative area of CRs and the latency to CR onset (including all eyeblink responses) across conditioning sessions, a statistically significant increase of relative area was observed from the 1st conditioning session (*P* < 0.05; Mann-Whitney test; Fig. 5d), and a significant latency decrease was also observed from the 1st conditioning session (*P* < 0.01; Mann-Whitney test, Fig. 5e). Interestingly, these significant differences were maintained for the peripheral-CS-induced CRs. In order to compare the characteristics of tACS-induced CRs and CRs evoked by peripheral whisker stimulation, we measured the relative area and the onset latency of both types of CRs. Regarding the CR relative area, no significant (*P* = 0.876; Mann-Whitney test) difference was found between CRs evoked by tACS stimulation of the SI (3.6 ± 0.2; n = 141) and those evoked by peripheral whisker stimulation (3.0 ± 0.1; n = 146). Likewise, no statistical significance was found in the onset latency of CRs when tACS (272.1 ± 2.6 ms; n = 141) or peripheral stimulation (271.1 ± 2.6 ms; n = 146) was used (*P* = 0.746; Mann-Whitney test). The values for relative area and onset latency for CRs collected from each conditioned animal are presented in Supplementary Table 3.

## Discussion

Over the last decade, transcranial stimulation of the brain by means of weak tDCS has been re-evaluated with notable success in both basic and clinical researchs^14^,^17^,^18^, and it is now known that tDCS is capable of inducing polarity-specific and long-lasting brain modulation non-invasively. More recently, the application of transcranial electrical stimulation with alternating currents, a technique derived from tDCS and called tACS, has been demonstrated to be a useful tool for exploring the functional role of oscillatory activities of the brain^27^,^28^,^29^,^30^,^31^,^32^,^33^. tACS also constitutes a novel approach for the exploration of perception processes and for sense synthesis at the cerebral cortical level^31^. Here, we show that 1) low-frequency tACS applied to the somatosensory cortex modifies the characteristics of LFPs in the SI vibrissa area of alert behaving rabbits in a polarity-dependent manner; 2) stimulation frequency determines the sub-threshold or supra-threshold character of tACS effects when applied over the motor cortex of the rabbit; and 3) tACS of the SI vibrissa area can train the animals for natural sensory stimulus during classical eyeblink conditioning.

Our results indicate that tACS applied over the somatosensory cortex at low frequencies (0.05 Hz) modulates the sensory input in an alternating manner. Thus, LFPs evoked by peripheral stimulation show an increase in the amplitude of the N1 component during anodal peaks of tACS, and a decrease during tACS cathodal troughs, with respect to control values. These results are in agreement with those reported during anodal and cathodal tDCS in the same rabbit preparation^25^,^35^. The changes in sensory LFPs in response to whisker stimulation during tACS reported here suggest a modification in the number of neurons recruited by the same stimulus, with consequent reinforcement or attenuation of the subjective perception of the stimulus^25^. According to its proposed modulatory effects, no direct motor output effects were observed when tACS was applied to the eyelid motor cortex at 0.05 Hz. The similarities observed in LFP modulation in response to simultaneous DC and slow AC transcranial stimulation suggest common underlying mechanisms and support previously reported polarity-dependent effects in the cortical excitability of humans^14^,^36^,^37^,^38^,^39^.

It is assumed that transcranial DC induces sub-threshold modulation of spontaneous cortical activity depending on neuronal morphology in the cortex and its compartment orientation with respect to the exogenous electric field direction^40^,^41^,^42^. The mechanisms underlying immediate DC effects are based on the redistribution of charges inside the neurons and its resultant membrane polarization in presence of exogenous electric fields^21^,^41^,^43^. Previous studies performed *in vitro* by using rat hippocampus slices show that changes induced by DC currents have a time constant of several tens of milliseconds, predicting weaker effects for AC fields^41^. This observation has been corroborated later in single hippocampal neurons (by using the same *in vitro* preparation), demonstrating that neuronal sensitivity to AC fields drops as an exponential decay function of frequency^44^. Nevertheless, the same study also shows that gamma oscillation (~30 Hz) induced by kainic acid perfusion in hippocampus slices shifted the frequency of the peak power from 31 Hz to 26 Hz, and substantially increased the power maximum (while reducing the power at the original peak), when a 50 Hz AC field was presented. This result suggests that emergent properties of neuronal networks, with a large number of interconnected neurons sharing common orientations in regard to the electric field, could drastically change neuronal sensitivity to alternating electric fields^44^. In a recent study, Ozen *et al*. (2010) demonstrated in anesthetized rats that alternating transcranial electrical stimulation at low frequencies (0.8–1.7 Hz) reliably entrained neurons in widespread cortical areas. The authors of the study suggest that sub-threshold electric fields can be effectively summed with sub-threshold network-induced membrane fluctuations generating spikes in a fraction of the neuronal population^34^. On the other hand, in the last few years, alternating-current stimulation has been demonstrated to interact with the human brain, enhancing sleep-associated consolidation of memory^45^, inducing visual phenomena^28^, enhancing individual alpha activity^30^, and increasing or inhibiting motor-cortex excitability^46^,^47^. Feurra *et al*. (2011) have shown that stimulation at alpha and high-gamma frequencies produces tactile sensation when tACS is applied over the human somatosensory cortex^31^. These results indicate that tACS interacts with the ongoing oscillatory cortical activity inducing supra-threshold effects (triggered neuronal spikes) in particular cerebral cortical areas at specific frequencies. The results in our study indicate that tACS applied over the eyelid motor cortex induces motor responses when applied at concrete frequencies (maintaining the same peak-to-peak amplitude and number of cycles), demonstrating that the sub-threshold or supra-threshold nature (either by direct trans-membrane potential disturbance or network resonance modulation) of tACS-associated effects depends on the specific frequency of the applied current. Interestingly enough, the maximal eyeblink amplitude response was observed at 30 Hz tACS, coinciding with beta-band frequency typically associated to motor commands in humans^48^.

After demonstrating that tACS over the motor cortex is able to trigger motor commands, we checked whether tACS applied over the SI was capable of inducing a tactile sensation. In the present paper we demonstrate that a short tACS pulse applied over the somatosensory cortex can be used successfully as a CS - i.e., it is able to evoke CRs similar to those evoked by a CS presented directly to the skin receptors. Thus, no differences in the learning rate and/or in some CR properties (total area, but not onset latency) were observed when peripheral stimulation or 30 Hz tACS were used as CS. In general, these results resemble those previously reported in rabbits demonstrating that direct intracortical stimulation of the somatosensory cortex can substitute for direct stimulation of whiskers during eyeblink conditioning^8^. In the latter paper, the authors convincingly demonstrate that kinematic properties of CRs were not modified by central vs. peripheral location of the CS. Interestingly, results collected when tACS stimulation at different frequencies was used as CS indicate that induction of an artificial sensation of whisker stimulation was more effective at 30 Hz and 100 Hz than at 10 Hz. Although all the tested frequencies were able to induce CRs, the analysis of the relative area and onset latency of these CRs highlighted important differences in their quality. This frequency-dependent effect of tACS has been reported previously in the human brain^28^,^29^,^30^,^31^,^32^,^33^,^45^,^46^,^47^. As alternating current applied over the brain cortex is postulated to interact with the ongoing cortical oscillatory activities, enhancing or diminishing them at specific frequencies, we decided to analyze the instant frequency of the different components constituting the sensory evoked potential by intracortical recording of the somatosensory cortex. This analysis shows that oscillation after the stimulus onset was close to ~80 Hz for the first two components, increasing to ~100 Hz for the remaining late components. These results are in agreement with the hypothesis that 100 Hz tACS applied over the somatosensory cortex may induce neuronal oscillation at natural frequencies for tactile sensation codification. In contrast, and based on the CR quantification, 10 Hz tACS application resulted in sensory perception (the subjects were able to respond in a conditioned manner) but differently to following the peripheral CS stimulus. Interestingly, no statistical difference was obtained when 30 Hz or 100 Hz tACS was presented as CS. Previous studies reporting tactile sensation by using tACS over the human somatosensory cortex also demonstrate that more than one frequency band was able to generate tactile sensation, with positive results obtained for the alpha-beta (10–20 Hz) and high-gamma (52–70 Hz) ranges^31^. In fact, when 100 Hz tACS was used in the rabbits, the values for relative area and onset latency of the CR improved with respect to those for 30 Hz tACS, appearing to substitute for tactile sensation in a more appropriate manner.

As expected from the above results, we demonstrated that animals can be entirely conditioned by using tACS-CS from the beginning of the eyeblink conditioning protocol and that once trained, were able to respond in a conditioned manner when peripheral sensory stimulus was presented for the first time. These results support the capability of tACS for inducing artificial sensations and constitute a proof of concept demonstration for tACS-based virtual-learning. Nevertheless, the reached asymptotic values of CR percentage for their learning curve was lower when tACS-CS was used (~70%) in comparison with asymptotic values obtained when peripheral whisker stimulation was used as CS (~90%). The diffuse spatial distribution of the transcranially applied electric field^25^,^49^ may underlie to the observed decrement in the learning curve. Intracortical microstimulation of SI cortex in animals has been previously performed for substitution of vibrissa stimulation during Pavlovian conditioning^8^, for perception of invisible light through coupling an infrared sensor output with the sensory cortex^12^, or for real-time transfer of sensorimotor information between the brains of two rats^11^. Although these studies call for a major role of sensory cortical prostheses in restoring neurological functions, their invasive nature seriously limits the use in human subjects. More recently, Grau and colleagues (2014) used robotized transcranial magnetic stimulation (TMS) for conscious transmission of information between human brains, providing a critical demonstration of brain-to-brain communication based in non-invasive technologies^15^.

Here we show electrophysiological and behavioral evidences supporting the use of non-invasive tACS for generating synthetic tactile sensations resembling natural ones. One of the main tACS limitations for its future application in human subjects is related with the low focality that characterizes this technique^49^. In this study, we use 4 small silver-ball electrodes instead of a bigger one for applying tACS in the large somatosensory region associated to whisker sensory inputs in the rabbit^50^. Interestingly, realistic modeling of electric fields in the human brain suggests that multifocal tCS devices using several small electrodes achieve more focal stimulation of specific targets than a large one^51^. It is expected that the potential use of non-invasive technologies such as TMS and tACS, will certainly play a role in the future application of computer-to-brain and brain-to-brain interactions in humans.

## Material and Methods

### Subjects

Experiments were carried out on adult rabbits (New Zealand White albino, from Isoquimen, Barcelona, Spain) weighing 2.3–2.7 kg upon arrival. Before and after surgery, animals were maintained in the same room, but placed in independent cages. Animals were kept on a 12 h light/dark cycle and with a continuous control of humidity (55 ± 5%) and temperature (21 ± 1 °C). Experimental procedures were carried out in accordance with European Union guidelines (2010/63/EU) and following Spanish regulations (RD 53/2013) for the use of laboratory animals in chronic experiments. Experiments were submitted to and approved by the local Ethics Committee of the Pablo de Olavide University (Seville, Spain).

### Surgery

Animals were anesthetized with a ketamine–xylazine mixture (Ketaminol, 50 mg/mL; Rompun, 20 mg/mL; and atropine sulfate, 0.5 mg/kg) at an initial dosage of 0.85 mL/kg. Anesthesia was maintained by i.v. perfusion at a flow rate of 10 mg/kg per h. A first group of animals (n = 5) was prepared for the chronic recording of LFPs evoked in the SI in response to whisker stimulation, without or in the presence of tACS. Under aseptic conditions, a hole (2 mm in diameter) was drilled in the parietal bone centered on the right SI vibrissa area (row C: AP = −1.7 mm, L = 7 mm^52^). The dura mater surface was protected with an inert plastic cover. In order to prepare a precisely focused active stimulating electrode^25^, four silver-ball electrodes (1 mm in diameter; A-M Systems, Everett, WA, USA) were symmetrically attached to the bone surface at 3 mm from the center of the drilled window (Fig. 1a) and covered with dental cement. A ground electrode (1 mm in diameter) in contact with the dura mater was attached to the left parietal bone (AP = 10 mm, L = 6 mm) as a reference for LFP recordings. A head-holding system, consisting of three bolts cemented to the skull perpendicularly to the stereotaxic plane, was also implanted. The stimulating electrodes were connected to a socket attached to the holding system.

A second group of animals (n = 3) was prepared for the recording of eyelid position and simultaneous tACS of motor cortex at different frequencies. A five-turn coil (3 mm in diameter) was implanted into the center of the left upper eyelid, close to the lid margin. Coils were made of Teflon-coated stainless-steel wire (A-M Systems, Everett, WA) with an external diameter of 50 μm. In order to stimulate the motor cortex region associated with eyelid movement, four silver-ball (1 mm in diameter; A-M Systems) stimulating electrodes were symmetrically attached to the bone surface at 2 mm from the right primary motor cortex (AP = −2 mm, L = 2 mm) and covered with dental cement. A head-holding system was implanted, and stimulating and recording electrodes were soldered to a socket attached to the holding system.

At last, a third group of animals (n = 6) was prepared for classical eyeblink conditioning and simultaneous tACS. Animals were implanted with recording bipolar hook electrodes in the left orbicularis oculi muscle. A pair of stimulating electrodes was implanted in the center of the whisker pad (row C, column 3). These electrodes were made of Teflon-coated stainless-steel wire (A-M Systems) with an external diameter of 230 μm and bared ~1 mm at the tip. Following the same procedure described above, four silver-ball (1 mm in diameter; A-M Systems) stimulating electrodes were symmetrically attached to the bone surface at 3 mm from the right SI vibrissa area and covered with dental cement. A head-holding system was implanted, and stimulating and recording electrodes were soldered to a socket attached to the holding system.

### Recording and stimulation procedures

Recording sessions began two weeks after surgery. Each animal was placed in a Perspex restrainer box designed for limiting animal movements^53^. The head of the animal was fixed to the recording table by means of the implanted head-holding system. For all subjects, the first two sessions consisted of adapting the animal to both restrainer and experimental conditions. For characterization of LFPs evoked in the somatosensory cortex, a glass micropipette was inserted into SI areas corresponding to the whiskers. The first recording sessions were used to map the receptor field of the contralateral whisker pad by air-puff stimulations of the whiskers. Once the SI was mapped, the glass micropipette was substituted by a chronically implanted Parylene-C® insulated tungsten microelectrode (0.5 MΩ resistance; A-M Systems). When located in the correct recording site, the tungsten electrode was attached to the skull, and the hole made in the parietal bone was covered with dental cement. LFPs were recorded using a Tektronix AM 502 differential amplifier with a bandwidth of 1 Hz to 10 kHz (Tektronix, Wilsonville, OR, USA). Eyelid movements were recorded with the magnetic field search coil technique as previously reported^53^. Along eyeblink conditioning sessions, the electromyogram (EMG) from the orbicularis oculi was recorded using a differential amplifier with a bandwidth of 1 Hz to 10 kHz (3600 model, A-M Systems). Air puffs directed at the whiskers, or the eye (during conditioning), were applied through the opening of a plastic pipette (3 mm in diameter) attached to a holder fixed to the recording table (air-puff device; Biomedical Engineering, Thornwood, NY, USA). Electrical stimulation of the whisker pad was achieved across an isolation unit (Cibertec, Madrid, Spain). Train stimuli (200 Hz) were programmed with the help of a CS-220 stimulator (Cibertec).

### Transcranial alternating-current stimulation

tACS was delivered by a battery-driven linear stimulus isolator (A395 Linear Stimulus Isolator; WPI, Sarasota, FL, USA). In rabbits, as opposed to humans, the skin overlying the cranium is highly movable with respect to the underlying bones. For this reason, tACS was applied simultaneously to the four implanted silver-ball electrodes, whilst a saline-soaked sponge (surface area = 35 cm^2^) attached to the contralateral ear served as counter electrode. To index cortical changes during tACS, LFPs evoked in response to whisker stimulation (air pulses, 100 ms, 2 kg/cm^2^, delivered every 10 ± 3 s) were recorded before (control) and during (immediate effects) tACS presentation. To characterize immediate effects, sine-wave current at 0.05 Hz and different intensities (1 mA, 2 mA, and 3 mA, peak-to-peak amplitudes) was applied separated by non-stimulation periods. In order to characterize anodal peak and cathodal trough direct-effects on sensory-induced LFP, air-puff stimulation of the contralateral whisker pad was triggered by peaks or troughs of the applied sine wave (Fig. 1b). To stimulate motor cortex a sine wave at intensities ranging from 2 to 3 mA and different frequencies (0.05 Hz, 0.1 Hz, 1 Hz, 10 Hz, 33 Hz, 100 Hz and 200 Hz) was applied separated by non-stimulation periods. Each stimulation frequency involved a 10-cycles sine wave and was repeated 5 times. During classical conditioning experiments, tACS was applied over the somatosensory cortex (100 ms in duration, 3–4 mA peak-to-peak amplitude) at 10 Hz, 30 Hz, and 100 Hz. According to the current distribution in a spherical head model^25^,^54^, the maximum current density in the stimulated brain was 3.7 A/m^2^ when 1 mA was applied.

### Classical eyeblink conditioning

Classical eyeblink conditioning was achieved with the help of a trace-conditioning paradigm. For this, animals were presented with a train of electrical stimuli (100 ms, 200 Hz) applied to the whisker pad as CS, followed 250 ms later by an air puff (100 ms, 3 kg/cm^2^) presented to the cornea as US. The CS applied to the whisker pad was presented on the same side (left) as the US. Conditioning sessions consisted of 66 trials (6 series of 11 trials each) separated at random by intervals of 50–70 s. Of the 66 trials, 6 were test trials in which the CS was presented alone. A conditioning session lasted ~80 min and each animal was trained for 10 successive days. The first two sessions consisted of the random presentation of CS alone (habituation sessions). As criteria, we considered a positive conditioned response (CR) the presence, during the CS-US interval, of EMG activity lasting >10 ms and initiated >50 ms after CS onset. In addition, the integrated EMG activity recorded during the CS-US interval had to be greater than the integrated EMG recorded immediately before CS presentation^8^,^53^,^55^. In order to characterize the impact of tACS on classical eyeblink conditioning, two additional conditioning sessions were carried out where the CS consisted of a tACS pulse (100 ms in duration, 3–4 mA peak-to-peak amplitude) applied over the SI at different frequencies. Thus, during the 11th conditioning session, whisker-pad stimulation (the initial CS) was substituted by tACS at 30 Hz, while for the 12th conditioning session whisker-pad stimulation was substituted by tACS at 10 Hz, 30 Hz, or 100 Hz (the new CS), distributed at random (22 stimuli for each frequency value).

Finally, with the aim of testing the potential use of tACS as CS during classical conditioning, eyeblink conditioning sessions were carried out in a separate group of animals where tACS (100 ms in duration, 3–4 mA peak-to-peak amplitude, 100 Hz) was used as CS during the first ten conditioning sessions. After training, during the 11th conditioning session, whisker pad stimulation was presented for the first time to the animals in order to test if peripheral tactile stimuli were able to induce CRs.

### Histology

At the end of the experiments, animals were deeply anesthetized with sodium pentobarbital (50 mg/kg, i.p.) and perfused transcardially with saline and 4% paraformaldehyde. The proper location of the whisker-pad stimulating and EMG recording electrodes was then checked. To confirm the final location of the electrodes implanted in the SI area, the brain was removed and cut into slices (50 μm), and the relevant cortical areas were processed for toluidine blue staining.

### Data collection and analysis

Sensory LFP and eyelid position recordings, tACS converted signals, unrectified EMG activity of the orbicularis oculi, and 1-V rectangular pulses corresponding to CS, US, and air-puff stimulations presented during the different experiments were stored digitally on a computer for quantitative off-line analysis (CED 1401-plus; CED, Cambridge, U.K.). Collected data were sampled at 20 kHz (for LFP recordings) or 10 kHz (for EMG recordings), with an amplitude resolution of 12 bits. A computer program (Spike2 from CED) was used for quantification. With the aid of cursors the peak amplitude and area of LFP evoked in the SI, and of onset latency, peak amplitude, and area of the rectified EMG activity of the orbicularis oculi muscle were measured.

Statistical analyses were carried out using the SPSS (SPSS Inc, Chicago, IL, USA) and SigmaPlot 11.0 (Systat Software Inc, San Jose, CA, USA) package. Statistical significance of differences between groups was inferred by one-way ANOVA and repeated-measures ANOVA. The nonparametric Mann–Whitney U test was applied for comparison when data did not permit normality assumption. Statistical significance was set at *P* < 0.05. The results are shown as mean ± SEM.

## Additional Information

**How to cite this article**: Javier, M.-R. *et al*. Synthetic tactile perception induced by transcranial alternating-current stimulation can substitute for natural sensory stimulus in behaving rabbits. *Sci. Rep.* **6**, 19753; doi: 10.1038/srep19753 (2016).

## Supplementary Material

Supplementary Information
