# Supplementary material for: Synthetic tactile perception induced by transcranial alternating-current stimulation can substitute for natural sensory stimulus in behaving rabbits
Source: Sci Rep. 2016 Jan 21;6:19753. doi: 10.1038/srep19753 (PMC4726368; doi:10.1038/srep19753)
Supplement: Supplementary Information [file srep19753-s1.doc]

**Synthetic tactile perception induced by transcranial alternating-current stimulation can substitute for natural sensory stimulus in behaving rabbits**

J. Márquez-Ruiz, C. Ammann, R. Leal-Campanario, G. Ruffini, A. Gruart, and J. M. Delgado-García

**Supplementary Tables**

| **Supplementary Table 1.** CR relative area and onset latency for each one of the participating animals during conditioning sessions 10 (C10) and 11 (C11), where whisker or tACS stimulus respectively was used as CS. | | | |
| --- | --- | --- | --- |
|  | **Subject** | **Conditioning session 10 (C10)** | **Conditioning session 11 (C11)** |
| **CR relative area** | #1 | 3.2±0.3 (n=57) | 3.6±0.3 (n=56) |
|  | #2 | 3.8±0.2 (n=62) | 2.4±0.1 (n=59) |
|  | #3 | 5.6±0.2 (n=65) | 5.6±0.2 (n=66) |
| **CR onset latency (ms)** | #1 | 272.1±4.6 (n=57) | 273.1±3.4 (n=56) |
|  | #2 | 268.7±3.2 (n=62) | 291.9±3.0 (n=59) |
|  | #3 | 230.6±3.2 (n=65) | 245.4±2.8 (n=66) |

| **Supplementary Table 2**. CR relative area and onset latency for each one of the participating animals during conditioning session 12 (C12), where tACS stimuli, randomly at 10 Hz, 30 Hz, and 100 Hz, were used as CS. | | | | |
| --- | --- | --- | --- | --- |
|  | **Subject** | **10 Hz** | **30 Hz** | **100 Hz** |
| **CR relative area** | #1 | 4.3±0.4 (n=19) | 7.5±0.6 (n=21) | 7.0±0.6 (n=22) |
|  | #2 | 2.7±0.3 (n=19) | 3.2±0.3 (n=21) | 3.9±0.3 (n=22) |
|  | #3 | 4.0±0.3 (n=22) | 4.7±0.4 (n=22) | 5.2±0.4 (n=22) |
| **CR onset latency (ms)** | #1 | 267.1±5.6 (n=19) | 245.4±4.8 (n=21) | 238.5±6.8 (n=22) |
|  | #2 | 291.0±5.7 (n=19) | 279.5±6.3 (n=21) | 257.8±4.7 (n=22) |
|  | #3 | 254.2±4.5 (n=22) | 243.4±6.0 (n=22) | 246.7±4.9 (n=22) |

| **Supplementary Table 3.** CR relative area and onset latency for each one of the participating animals during conditioning sessions 10 (C10) and 11 (C11), where tACS or whisker stimulus respectively was used as CS. | | | |
| --- | --- | --- | --- |
|  | **Subject** | **Conditioning session 10 (C10)** | **Conditioning session 11 (C11)** |
| **CR relative area** | #1 | 5.7±0.4 (n=58) | 3.8±0.2 (n=58) |
|  | #2 | 1.7±0.1 (n=41) | 2.4±0.1 (n=48) |
|  | #3 | 2.5±0.2 (n=42) | 2.7±0.2 (n=40) |
| **CR onset latency (ms)** | #1 | 252.0±3.9 (n=58) | 271.3±3.4 (n=58) |
|  | #2 | 287.5±3.8 (n=41) | 265.7±5.1 (n=48) |
|  | #3 | 284.9±3.5 (n=42) | 277.5±5.3 (n=40) |
